# SMAD transcription factors are altered in cell models of HD and regulate *HTT* expression

**DOI:** 10.1016/j.cellsig.2016.12.005

**Published:** 2017-02

**Authors:** KR Bowles, T Stone, P Holmans, ND Allen, SB Dunnett, L Jones

**Affiliations:** a The MRC Centre for Neuropsychiatric Genetics and Genomics, Cardiff University School of Medicine, Hadyn Ellis Building, Maindy Road, Cathays, Cardiff CF24 4HQ, UK; b Cardiff School of Biosciences, The Sir Martin Evans Building, Museum Avenue, Cardiff CF10 3AX, UK; c The Brain Repair Group, School of Biosciences, Cardiff University, Museum Avenue, Cardiff CF10 3AX, UK

**Keywords:** Huntington's Disease, Transcription, EGF, TGF-beta, SMAD, Kinase signalling

## Abstract

Transcriptional dysregulation is observable in multiple animal and cell models of Huntington's disease, as well as in human blood and post-mortem caudate. This contributes to HD pathogenesis, although the exact mechanism by which this occurs is unknown. We therefore utilised a dynamic model in order to determine the differential effect of growth factor stimulation on gene expression, to highlight potential alterations in kinase signalling pathways that may be in part responsible for the transcriptional dysregulation observed in HD, and which may reveal new therapeutic targets. We demonstrate that cells expressing mutant huntingtin have a dysregulated transcriptional response to epidermal growth factor stimulation, and identify the transforming growth factor-beta pathway as a novel signalling pathway of interest that may regulate the expression of the *Huntingtin (HTT)* gene itself. The dysregulation of *HTT* expression may contribute to the altered transcriptional phenotype observed in HD.

## Highlights

• Dysregulation of TGFβ signalling is identified in mouse and human cell models of Huntington's disease.
• Characterisation of this dysregulation in downstream SMAD transcription factors is presented.
• SMAD3 binds to the *HTT* promotor and can regulate *HTT* expression in the absence of a CAG expansion.

## 1 Introduction

Huntington's Disease (HD) is an autosomal dominant neurodegenerative disorder caused by a CAG expansion within the first exon of the *Huntingtin* (*HTT)* gene, which gives rise to an expanded polyglutamine tract in the huntingtin (HTT) protein. HD is characterised by progressive motor abnormalities that manifest in the third to fourth decades of life, and is also commonly associated with cognitive impairments and psychiatric disturbances [1]. Neuronal dysfunction has been found to occur prior to both striatal atrophy and overt motor symptom onset [2], [3]. It is therefore possible that cell death and degeneration in HD-affected neuronal cells follow an initial period of dysregulation of multiple cellular processes [4].

The regulation of kinase signalling is altered by, and in turn alters, gene expression: in HD aberrant regulation of multiple kinase signalling pathways has been shown [5]. The TGFβ pathway is a regulator of cell growth, proliferation and apoptosis, and is upstream of the core regulatory mothers against decapentaplegic-homolog (SMAD) family of transcription factors [6], [7]. To date, the characterisation of TGFβ1 in association with HD has been limited, and has yielded contradictory results; TGFβ1 is reduced in the peripheral blood of asymptomatic HD patients, and is inversely correlated with CAG repeat length [8]. However, while YAC128 and R6/2 mice exhibit reduced TGFβ1 in the cortex, increased TGFβ1 has been observed in HD patient and R6/2 mouse plasma [9]. Increased TGFβ signalling has also been identified in the hippocampus of a transgenic rat model of HD and in the R6/2 mouse model, where it has an inverse effect on neural stem cell proliferation [10], and in the cortex of the Q175 mouse model [11]. The TGFβ pathway is upregulated in human HD induced pluripotent stem cells (hiPSCs) and restored to normal levels by replacement of the expanded CAG repeat with a CAG repeat of non-pathogenic length [12]. Further analysis of iPSC-derived neural progenitor cells (NPCs) carrying expanded CAG repeats showed increased levels of TGFβ1 and enhanced SMAD2 phosphorylation [11].

We investigated differential gene expression after epidermal growth factor (EGF) stimulation in the immortalised *StHdh*^*Q111*^ cell model of HD and identified TGFβ signalling as a dysregulated pathway. Further characterisation of this pathway in both the *StHdh*^*Q111*^ model and in hiPSC-derived NPCs revealed dysregulation of SMAD expression, localisation and phosphorylation in cells carrying a CAG expansion, as well as evidence of direct regulation of *Htt* gene expression by Smad3 activation.

## 2 Methods

### 2.1 Cellular models

*StHdh*^*Q7/7*^, *StHdh*^*Q7/111*^ and *StHdh*^*Q111/111*^ immortalised embryonic striatal cells were a kind gift from Marcy MacDonald (Molecular Neurogenetics Unit, Massachusetts General Hospital, Massachusetts, USA). Cell lines were grown and maintained in high glucose Dulbecco's modified eagle medium (DMEM; Life Technologies), containing 1% penicillin-streptomycin solution, 1% 40 mg/ml Geneticin (both Life Technologies) and 10% fetal bovine serum (FBS; PAA), in a humid environment at 33 °C with 5% CO_2._

**Supplementary Fig. 1:**
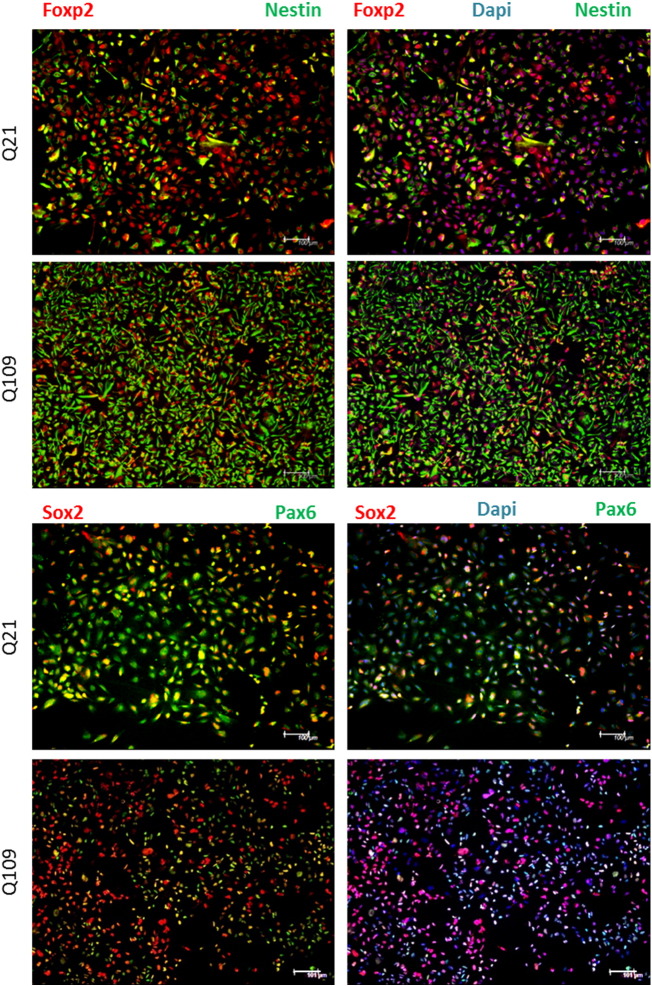
Characterisation of Q21 and Q109 iPSC-derived neural progenitor cells. Both lines express the neuronal pluripotency markers FOXP2, NESTIN, SOX2 and PAX6, as well as a DAPI nuclear stain. Both cell lines exhibit slightly different morphology; Q21s are generally larger than Q109 cells. However, this is similar to the heterozygote *StHdh*^*Q7/111*^ cells in comparison to wild-type *StHdh*^*Q7/7*^ cells so may be a result of *mHTT* expression. Images are representative of multiple images taken from 3 separate coverslips per condition. Scale bar = 100 μM

Q109 (heterozygous for a 109 CAG repeat expansion) and Q21 (wild-type, homozygous for 21 CAG repeats) hiPSC-derived lines were maintained at the NPC stage of differentiation, in order to best match the immortalised *StHdh*^*Q111*^ cell lines. NPCs were grown and maintained on Matrigel-coated plates (VWR) in Expansion media consisting of advanced DMEM F12, supplemented with 1% penicillin-streptomycin solution, 1% glutamine supplement (all Life Technologies), 10 μg/ml epidermal growth factor (EGF; Peprotech), 10 μg fibroblast growth factor (FGF; Peprotech) and 2% Neurobrew with vitamin A (Miltenyi). Cells were grown in a humid environment at 37 °C with 5% CO2. Media was replaced daily, and cells were passaged upon reaching 90% confluence using Accutase (Life Technologies). Immunofluorescence was carried out on these cells with common NPC markers to confirm differentiation stage (Supplementary Fig. 1).

### 2.2 Growth factor stimulation

Cells were serum starved overnight prior to treatment with EGF (Life Technologies). Following serum starvation, cells were incubated for 2 h with 100 ng/ml EGF, followed immediately by RNA extraction. Control cells were serum starved for the same period of time and processed in parallel with EGF treated cells.

In order to induce Smad phosphorylation, cells were serum starved overnight, then incubated with 100 ng/ml of either mouse (for *StHdh*^*Q111*^ cells) or human (for hiPSC-derived NPCs) TGFβ1 (NEB) for 30 min (for protein analysis) or for 2 h (for nucleic acid analysis).

### 2.3 RNA extraction

RNA was extracted from cells grown in 6-well plates using the phenol/chloroform method, precipitated in ethanol, and purified using RNeasy MinElute Cleanup kit (Qiagen) according to manufacturer's instructions.

### 2.4 Microarray and bioinformatics analysis

The microarray was kindly carried out by Cardiff University's Central Biotechnology Services (CBS), utilising the Affymetrix GeneChip Mouse Gene 1.0 ST array system.

Raw microarray data was analysed in Partek® Genomics Suite™, where it underwent quantile normalisation to log base 2 and median polish probeset summarisation. There was no adjustment for GC content or probe sequence, and robust multi-array average (RMA) background correction was applied. Gene expression data were analysed by a 2-way ANOVA containing a ‘genotype x EGF’ interaction term to identify genes for which EGF stimulation resulted in significantly different expression changes between the genotypes, plus individual contrasts to identify simple main effects, that is, genes for which EGF stimulation significantly changes expression in a particular genotype. The focus for the following stages of analysis was on the differences between *StHdh*^*Q7/7*^ and *StHdh*^*Q111/111*^ cell lines only, as this is where the PCA suggested the largest differences are likely to lie. The following lists of significantly differentially expressed genes were created from normalised data with the parameters described in Supplementary Table 1. Individual gene lists for differentially expressed genes following EGF stimulation in *StHdh*^*Q7/7*^ and *StHdh*^*Q111/111*^ cells were created. As there were a large number of significant genes in the genoype x EGF interaction list, only the top 500 most significant genes were selected in order to focus on the genes with the strongest effect. These lists were put through pathway analyses using The Database for Annotation, Visualization and Integrated Discovery (DAVID) v6.7 (http://david.abcc.ncifcrf.gov/; [13], [14]). To widen the analysis beyond the regulation of transcription, all of the enriched functional terms from the DAVID analysis were subject to additional clustering to eliminate redundancy and to clarify visualization.

Clustering of terms showing significant enrichment from the DAVID analysis was performed as follows: Firstly, the terms were ranked in order of enrichment significance, with the most significant term first. For each term in turn, a measure of overlap was calculated between that term and the terms in each of the existing clusters. The measure of overlap used was the Jaccard index, calculated as the number of genes associated with both terms divided by the number of genes associated with either term. The term was assigned to the cluster with which it had the highest average Jaccard index, provided this was > 50%. If the term did not have an average Jaccard index > 50% with any existing cluster, it was placed in a new cluster. This process was repeated until the end of the list of terms was reached. The resulting clusters were visualised in Cytoscape [15].

### 2.5 qRTPCR

TaqMan (Life Technologies) qRTPCR technologies were utilised throughout this project using a two-step protocol. Extracted RNA was reverse transcribed to cDNA using the High Capacity RNA-to-cDNA kit (Life Technologies) according to manufacturer's instructions, prior to quantitative PCR utilising pre-designed TaqMan gene expression assays and Fast Advanced Master Mix (Life Technologies) according to manufacturer's instructions.

### 2.6 Western blotting

Protein lysates were extracted from *StHdh*^*Q111*^ and NPC lines using 10 × Cell Lysis Buffer (NEB) according to manufacturer's instructions. 20 μg protein from each sample was resolved on a 4–12% Bis-Tris gel (Life Technologies) and electroblotted onto a PVDF membrane (Millipore). Blots were incubated for 30 min in 5% skimmed milk powder in PBS with 0.1% Tween (PBS-T), and were then incubated overnight at 4 °C with the appropriate primary antibody. The next day, blots were washed with PBS-T, and incubated with the appropriate peroxidase secondary antibody (Vector Laboratories) for 1 h at room temperature. Bands were then detected using an enhanced chemiluminescent HRP substrate (SuperSignal West Dura Extended Duration Substrate; Fisher Scientific), and visualised after exposure onto Hyperfilm (Fisher Scientific) and processing through an Ecomax X-ray film processor. Processed blots were then stripped using Restore PLUS stripping buffer (Fisher Scientific), probed with additional antibodies and re-imaged. Blots were stripped and re-probed up to a maximum of 4 times before being discarded.

### 2.7 Immunofluorescence

Cells were grown on glass coverslips and fixed by incubation with 10% Formalin solution (Sigma Aldrich). They were then permeabilised with 0.1% Triton x-100 (Life Technologies) in PBS, and blocked with 1% (*w*/*v*) bovine serum albumin (BSA; Sigma Aldrich) in PBS. Cells were then incubated with the relevant primary antibody for 1 h in a humid environment at 37 °C, followed by the appropriate AlexaFluor secondary antibody (LifeTechnologies) for 1 h in a humid environment at 37 °C, while wrapped in foil. Coverslips were mounted onto glass microscope slides using ProLong Gold antifade reagent with DAPI (Life Technologies), and stored in the dark at 4 °C for at least 24 h before visualization. Slides were imaged on a Leica DM6000B fluorescent microscope and nuclear/cytoplasmic mean pixel intensity ratios were calculated as previously described [16]

### 2.8 Antibodies

See Supplementary Table 2.

### 2.9 Chromatin immunoprecipitation

ChIP was carried out using approximately 4 × 10^7^ cells per condition, grown in multiple 15 cm dishes. Prior to chromatin preparation, DNA was crosslinked using formaldehyde and glycine. Chromatin preparation, digestion and immunoprecipitation were carried out using the SimpleChIP kit (NEB), according to manufacturer's instructions. The antibodies used for chromatin immunoprecipitation were *anti*-SMAD3 (1:50; NEB) and anti-phosphorylated SMAD3 (1:100; NEB). Histone *H*3 (1:50) and normal rabbit IgG (1 μg) were used as negative controls (provided in SimpleChIP kit). The resulting precipitated DNA was amplified using qPCR and primers against the 5′ end of the *Htt* gene, incorporating the SBE region. Both antibodies were compared against normal rabbit IgG. Purified DNA from the ChIP process was quantified by Sybr Green (Life Technologies) qPCR using the following conditions; a. initial denaturation 95 °C 3 min, b. denaturation 95 °C 15 s, c. anneal and extension 60 °C 60 s, d. repeat b and c for a total of 40 cycles. qPCR primers were designed to incorporate the Smad binding element (SBE) in the 5′ upstream promoter region of the Htt gene, and were as follows; forward 5’CTGAGCGCCTTGGTTCCG-3′, reverse 5′- ATCAGCTTTTCCAGGGTTGC-3′.

### 2.10 SMAD inhibition

In order to inhibit SMAD phosphorylation, cells were serum starved overnight, and then incubated with 5uM of the SMAD2/3 inhibitor SB525334 (Millipore) for 1 h prior to stimulation with 100 ng/ml TGFβ1, and collection of either protein lysates or extraction of RNA. Incubation with the equivalent volume of DMSO was used for control conditions.

### 2.11 Statistical analyses

For qRTPCR analyses, delta-Ct values were subject to a 2-way ANOVA, followed by post-hoc Tukey HSD tests. Western blots were semi-quantitatively analysed using densitometry analysis in ImageJ. The resulting measurements were normalised to measurements of α-tubulin, and subject to the appropriate ANOVA and post-hoc Tukey tests. ChIP qPCR data was analysed using the fold-enrichment method; Ct values were normalised to the 2% input control, and the resulting fold-change as compared to the IgG control was determined. The resulting data was subject to a 2-way ANOVA and post-hoc Tukey tests.

## 3 Results

### 3.1 Differential gene expression implicates TGFβ signalling pathways in mutant *HTT* carrying cells

**Supplementary Fig. 2:**
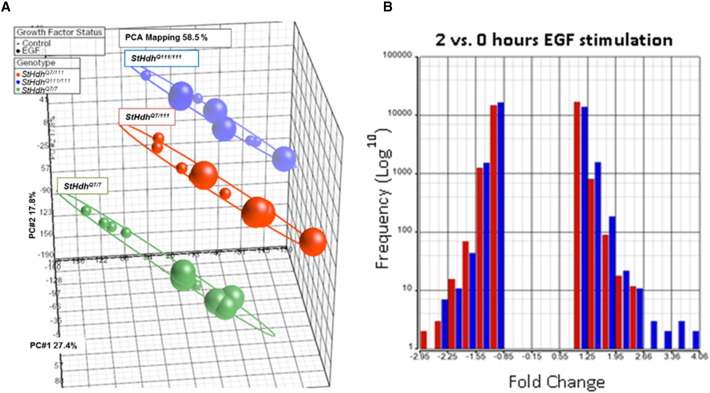
Summary of EGF microarray data. **A.** PCA of EGF microarray data. Genotype is the main principle component, accounting for 27.4% of gene expression variance. EGF stimulation is the second principle component, and accounts for 17.8% of the variance. Each genotype clusters together, but there is clear separation between 0 and 2 h EGF stimulation in *StHdh*^*Q7/7*^ cells (green). This separation is reduced in the *StHdh*^*Q7/111*^ cells (red), and is not apparent in the *StHdh*^*Q111/111*^ cells (blue). Baseline = small spheres, EGF stimulation = large spheres (*n* = 5). More genes were differentially expressed after EGF stimulation in *StHdh*^*Q7/7*^ cells (391) compared with *StHdh*^*Q111/111*^ cells (254), and 2687 genes exhibited a significant interaction between genotype and EGF stimulation. **B.** Histogram of the frequency of the magnitude of gene expression fold changes between 0 and 2 h stimulation for both *StHdh*^*Q7/7*^ (blue) and *StHdh*^*Q111/111*^ (red) cells. Both *StHdh*^*Q7/7*^ and *StHdh*^*Q111/111*^ cells exhibit similar distributions of gene expression fold changes in response to EGF, and the majority of changes cluster between − 4 and + 4. Skew = − 0.17 (*StHdh*^*Q7/7*^), − 0.09 (*StHdh*^*Q111/111*^) & EGF kurtosis = − 1.9 (*StHdh*^*Q7/7*^), − 1.93 (*StHdh*^*Q111/111*^). *StHdh*^*Q7/7*^ cells have a larger range of positive fold changes following EGF stimulation than *StHdh*^*Q111/111*^ cells, concordant with a larger transcriptional effect of EGF also evident from the PCA (***A***).

We detected substantial differences in gene expression between *StHdh*^*Q7/7*^, *StHdh*^*Q7/111*^ and *StHdh*^*Q111/111*^ cell lines in response to EGF treatment (Supplementary Fig. 2, Supplementary Table 3) with genotype being the main source of variation, and stimulation with EGF the second largest source. There was also a *mHTT/HTT* gene dosage effect on expression. As there were substantial differences in gene expression profiles between *StHdh*^*Q7/7*^ and *StHdh*^*Q111/111*^ cells at baseline, we compared the extent of gene expression changes following EGF treatment within each cell line, rather than directly comparing levels of gene expression between the two genotypes.

Similar GO terms were implicated following EGF stimulation in both *StHdh*^*Q7/7*^ and *StHdh*^*Q111/111*^ cell lines (Supplementary Table 4). Although some pathways were associated with developmental processes, the majority of the most significant pathways were related to transcription and transcriptional control. The Transcription and Positive Regulation of Transcription pathways were also amongst the most significant pathways derived from genes showing differential effects of EGF stimulation between cell lines.

**Fig. 1:**
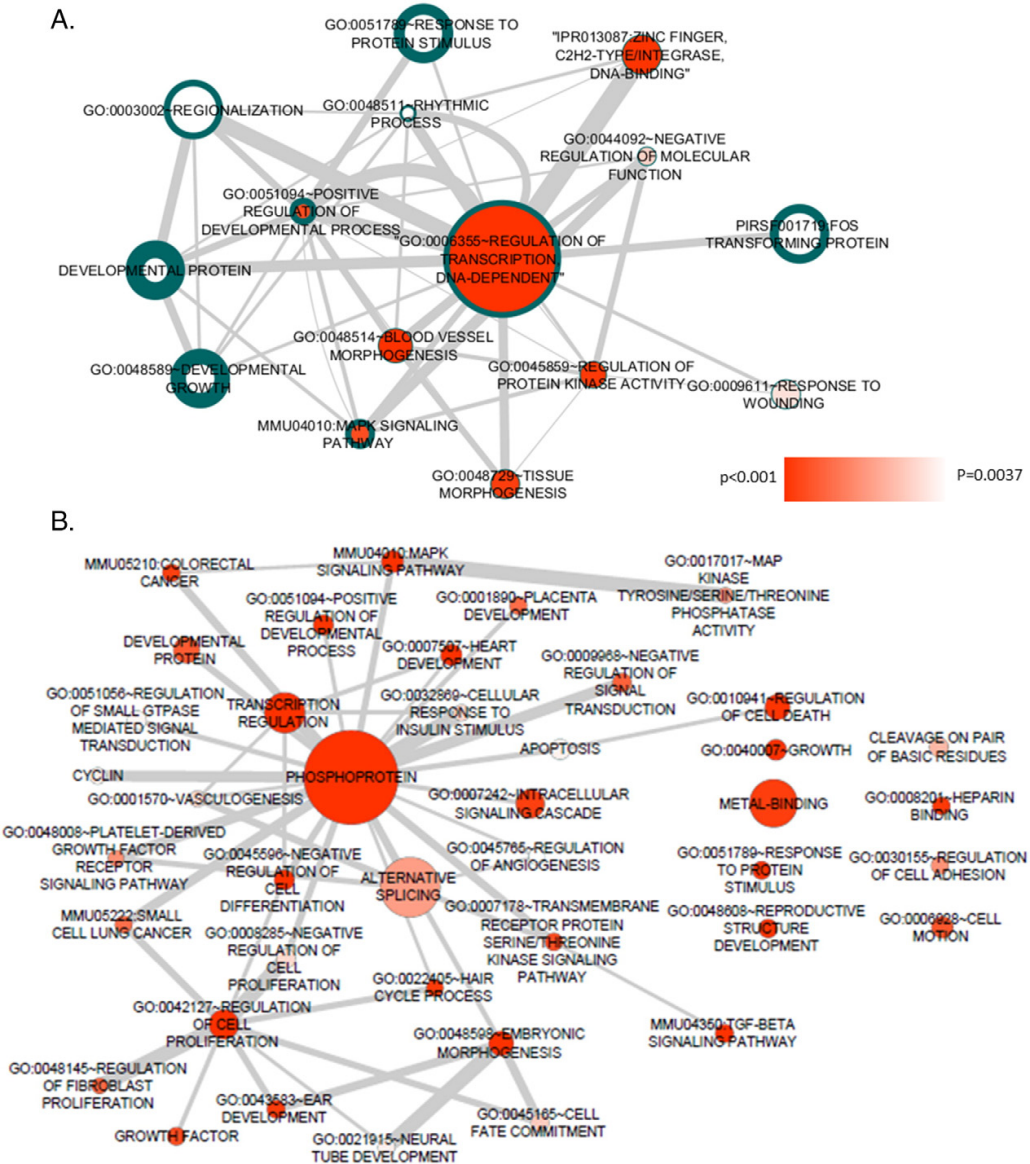
Functional annotation networks of differential gene expression following EGF stimulation. ***A.****StHdh*^*Q7/7*^ and *StHdh*^*Q111/111*^ DAVID functional annotations for differential gene expression following EGF stimulation, following our clustering algorithm and visualised together as pathway networks in Cytoscape. Node colour saturation is indicative of *StHdh*^*Q7/7*^ cluster *p*-value, and the width of node outlines indicates *StHdh*^*Q111/111*^ cluster *p*-values (thicker outlines correspond to smaller *p*-values). Node size positively correlates with the size of the gene set contributing to *StHdh*^*Q7/7*^-associated nodes. Edge width positively correlates with the similarity coefficient between the two connected nodes. ***B***. DAVID functional annotations following our clustering algorithm for the interaction term gene list, visualised as pathway networks in Cytoscape. Node colour saturation is indicative of cluster *p*-value, node size positively correlates with the size of the gene set contributing to the node. Edge width positively correlates with the similarity coefficient between the two connected nodes.

We clustered our DAVID pathway analysis outputs to simplify pathway enrichments without compromising the diversity of terms by eliminating terms with overlapping genes (see Section 2, Methods). Clustered nodes were then visualised as a network of functional categories using Cytoscape (Fig. 1;(15)). The Regulation of Transcription, DNA-dependent node contained the largest number of differentially expressed genes in both genotypes and was significantly enriched (*p* = 8.13 × 10^12^). A comparison between *StHdh*^*Q7/7*^ and *StHdh*^*Q111/111*^ EGF-stimulated networks indicated minimal overlap between pathway nodes for each genotype (Fig. 1), although Positive Regulation of Developmental Processes, Rhythmic Processes and MAPK Signalling were significant for both cell lines (Table 1).

The same clustering was applied to a list of genes with a significant interaction between the effect of growth factor stimulation and genotype (Fig. 1; Table 1). At the centre of the network is a large Phosphoprotein node consisting of 228 genes. The next two largest nodes are Transcription Regulation and Alternative Splicing, which each share 66% of their gene set with the larger Phosphoprotein node. TGFβ Signalling was a significantly enriched term resulting from the investigation of differentially expressed genes between *StHdh*^*Q7/7*^ and *StHdh*^*Q111/111*^ cells following EGF stimulation (Fig. 1; Table 1). Regulatory genes within this pathway, such as *Smad3* and *Smad7*, were also present within the larger, and more central Phosphoprotein node. We therefore followed this up and investigated TGFβ signalling in HD cell models.

### 3.2 Alterations in TGFβ signalling

**Supplementary Fig. 3:**
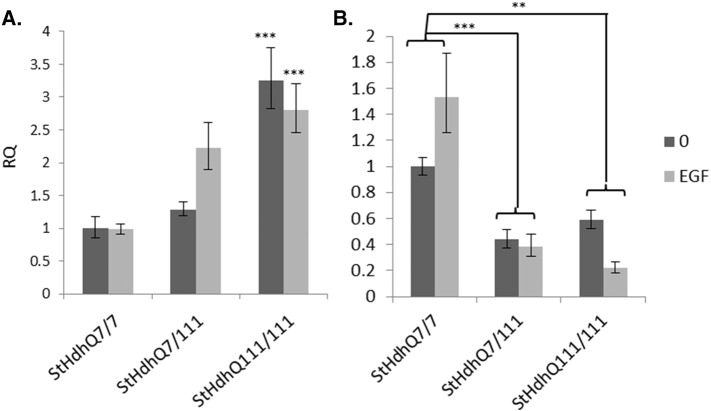
*Smad3* and *Smad7* expression. RQ values for ***A***. *Smad3* and ***B.****Smad7* expression in *StHdh*^*Q111*^ cell lines following 2 h stimulation with 100 ng/ml EGF. Asterisks denote a significant difference from *StHdh*^*Q7/7*^ cells unless otherwise marked. *** *p* < 0.001. *N* = 6.

One of the inhibitory members of the TGFβ signalling family, *Smad6*, was identified and validated as a gene of interest (Supplementary Table 3). Both the activating *Smad3* and inhibitory *Smad7* exhibited similar patterns of expression to the microarray data, as detected by qRTPCR, and the effect of genotype on their expression at baseline was validated (Supplementary Fig. 3).

Altered expression of additional members of the TGFβ signalling pathway, such as *Smads 1*, *2*, *5*, and TGFβ receptors, was also observed in *StHdh*^*Q111/111*^ cells within the microarray data, supporting the hypothesis that this pathway is dysregulated in these cells (Table 2).

### 3.3 Characterisation of SMAD expression and activity in *StHdh*^*Q111*^ and hiPS-derived neural progenitor cells

In order to characterise the TGFβ signalling pathway, we used TGFβ1 as a stimulus.

**Fig. 2:**
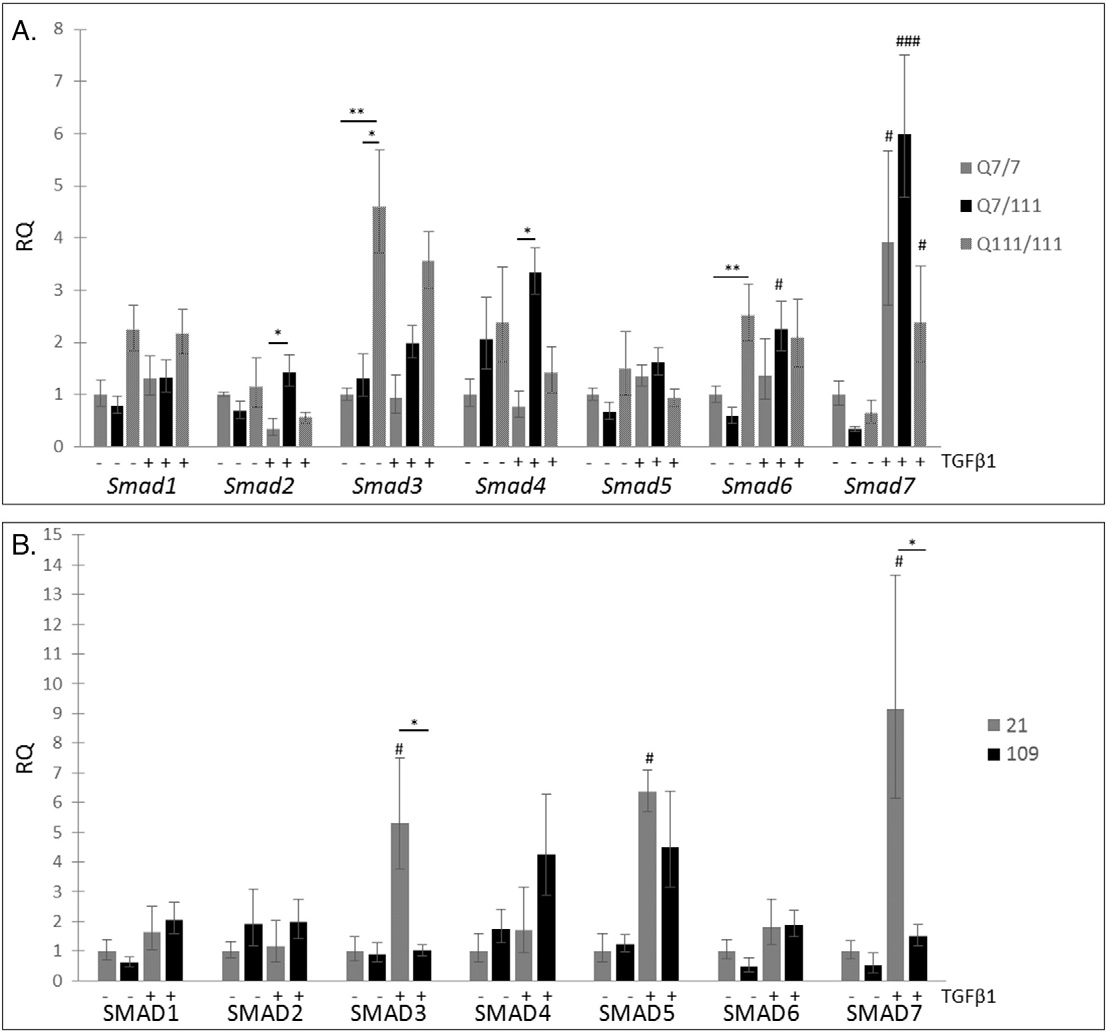
SMAD expression in mouse *StHdh* cell lines and human NPC cells. RQ values for *Smad* TF expression in ***A***. *StHdh*^*Q111*^ cell lines and ***B.*** hiPSC-derived NSCs measured by qRTPCR both prior to, and following 2 h stimulation with 100 ng/ml TGFβ1. * *p* < 0.05, ** *p* < 0.01, *** *p* < 0.001. Asterisks denote a significant difference between cell lines, hashes denote significant differences within cell lines. *N* = 6 for each *StHdh*^*Q11*1^ comparisons, and NSC comparisons. In *StHdh*^*Q111*^ cells (***A***), there was an effect of both genotype (F2, 30 = 4.066, *p* < 0.05) and TGFβ1 (*F*1,30 = 4.814, *p* < 0.05) on the expression of *Smad6*, as well as a significant genotype x TGFβ1 interaction (F2,30 = 4.076, *p* < 0.05). There was also a significant effect of TGFβ1 on *SMAD6* expression in Q21 and Q109 cells (***B***; *F*1,17 = 7.548, *p* < 0.05), with the fold change increase being larger in Q109 cells (× 1.8 Q21 vs. × 3.8 Q109). *Smad7* expression in *StHdh*^*Q111*^ cells was increased by TGFβ1 stimulation (**A**; *F*1,30 = 59.295, *p* < 0.001), and showed a significant genotype x TGFβ1 interaction (F2,30 = 4.415, *p* < 0.05). *SMAD7* expression in Q21 and Q109 cells (***B***) also had a significant effect of both genotype (*F*1,17 = 8.483, *p* < 0.05) and TGFβ1 (*F*1,17 = 14.927, *p* < 0.01).

There was little effect of TGFβ1 stimulation on the expression of *Smads 1,2,4* and *5* in mouse *StHdh*^*Q111*^ cell lines or in the human iPSC-derived NPCs, although multiple alterations in expression were seen between *StHdh*^*Q7/7*^, *StHdh*^*Q7/111*^ and *StHdh*^*Q111/111*^ cells both prior to, and following TGFβ1 stimulation, which most commonly exhibited a trend towards increased expression in *mHtt*-carrying cell lines (Fig. 2). *Smad3* expression was significantly higher in *StHdh*^*Q111/111*^ cells at baseline, compared with both *StHdh*^*Q7/7*^ (*p* < 0.001) and *StHdh*^*Q7/111*^ cells (*p* < 0.05), whereas significantly higher levels of *SMAD3* expression were observed in the human Q21 iPSC-derived NPCs compared to the human Q109 iPSC-derived NPCs (*p* < 0.05). The expression of *Smad6* and *Smad7* were both altered by TGFβ1 stimulation in both mouse *StHdh*^*Q111*^ and human NPC lines (Fig. 2); there was increased expression of *Smad6* when homozygous *Q*^*111*^ alleles were present in the absence of TGFβ1, and in *StHdh*^*Q7/111*^ cells in the presence of TGFβ1. Human Q21 and Q109 cells also exhibited increased *SMAD6* expression in response to TGFβ1. TGFβ1 stimulation increased *Smad7* expression in all mouse cell lines independent of genotype, and the most substantial effect of TGFβ1 in the human iPSC lines was on *SMAD7* expression*.* Despite the Q109 NPC line being heterozygote for the *mHTT expansion*, the expression of *SMAD7* mirrors that observed in the homozygous *StHdh*^*Q111/111*^ line. Similar to the *StHdh*^*Q7/7*^ cells, there was a significant effect of TGFβ1 in control Q21 cells (*p* < 0.05), however this effect was suppressed in Q109 cells, which expressed significantly less *SMAD7* compared to Q21 cells following growth factor stimulation (*p* < 0.05).

### 3.4 Aberrant localisation of SMAD proteins in mouse *StHdh*^*Q111*^ and human NPC lines

**Fig. 3:**
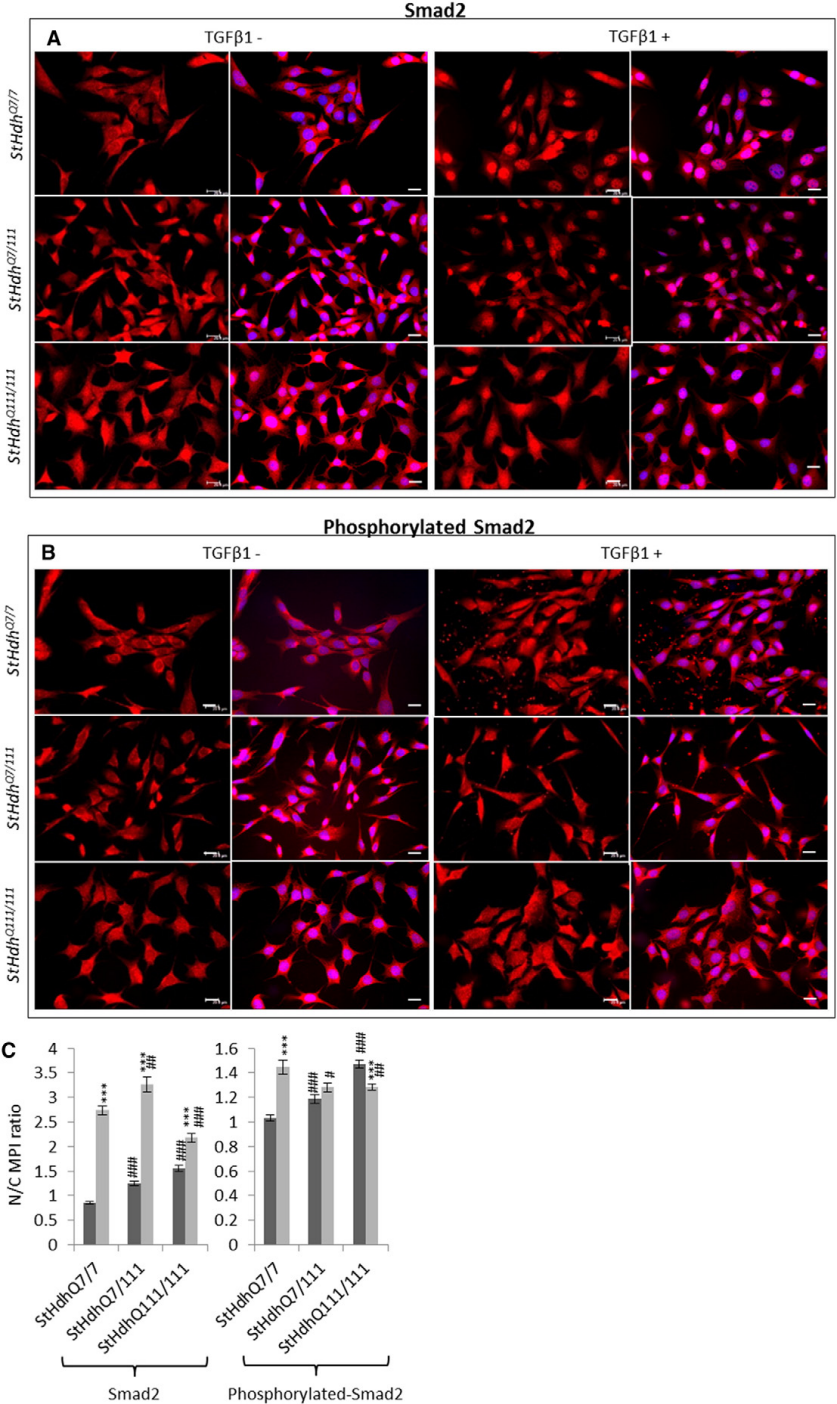
Total and phosphorylated Smad2 localisation in mouse *StHdh* cell lines. Fixed *StHdh*^*Q7/7*^, *StHdh*^*Q7/111*^ and *StHdh*^*Q111/111*^ cells stimulated with either 0 or 100 ng/ml TGFβ1 for 30 min, stained with antibodies against **A**. Smad2 and **B**. phosphorylated Smad2, with nuclei counterstained with DAPI (blue). Images are representative of multiple replications. Scale bar = 20 μM. Figure representative of multiple images, *N* = 3. **C**. Quantification of the nuclear/cytoplasmic mean pixel intensity (N/C MPI) ratio of the experiments presented in ***A*** and ***B***, based on an average of 90 cells per condition, taken from 6 different frames across 3 experiments (see Section 2, Methods). Asterisks denote a significant difference from 0 ng/ml TGFβ, hashes indicate a significant difference from *StHdH*^*Q7/7*^ cells. */# *p* < 0.05, **/## *p* < 0.01, ***/### *p* < 0.001.

**Fig. 4:**
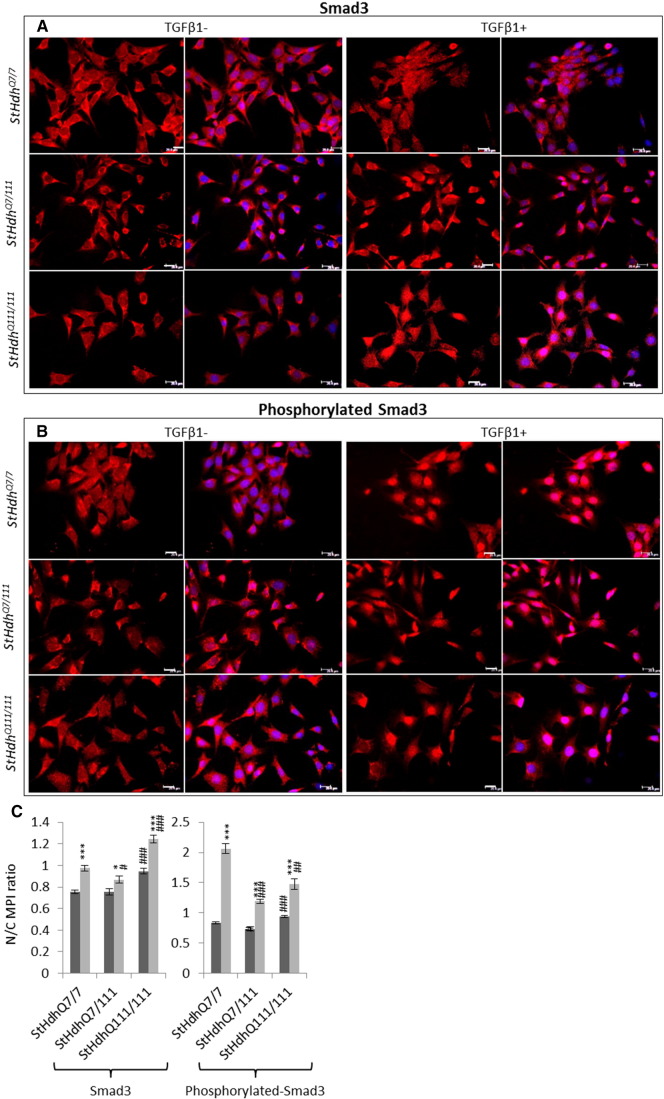
Total and phosphorylated Smad3 localisation in mouse *StHdh* cell lines. Fixed *StHdh*^*Q7/7*^, *StHdh*^*Q7/111*^ and *StHdh*^*Q111/111*^ cells stimulated with either 0 or 100 ng/ml TGFβ1 for 30 min, stained with antibodies against A. Smad3 and B. phosphorylated Smad3, with nuclei counterstained with DAPI (blue). Images are representative of multiple replications. Scale bar = 20 μM. Figure representative of multiple images, *N* = 3. **C**. Quantification of the nuclear/cytoplasmic mean pixel intensity (N/C MPI) ratio of the experiments presented in ***A*** and ***B***, based on an average of 90 cells per condition, taken from 6 different frames across 3 experiments (see Section 2, Methods). Asterisks denote a significant difference from 0 ng/ml TGFβ, hashes indicate a significant difference from *StHdH*^*Q7/7*^ cells. */# *p* < 0.05, **/## *p* < 0.01, ***/### *p* < 0.001.

**Fig. 5:**
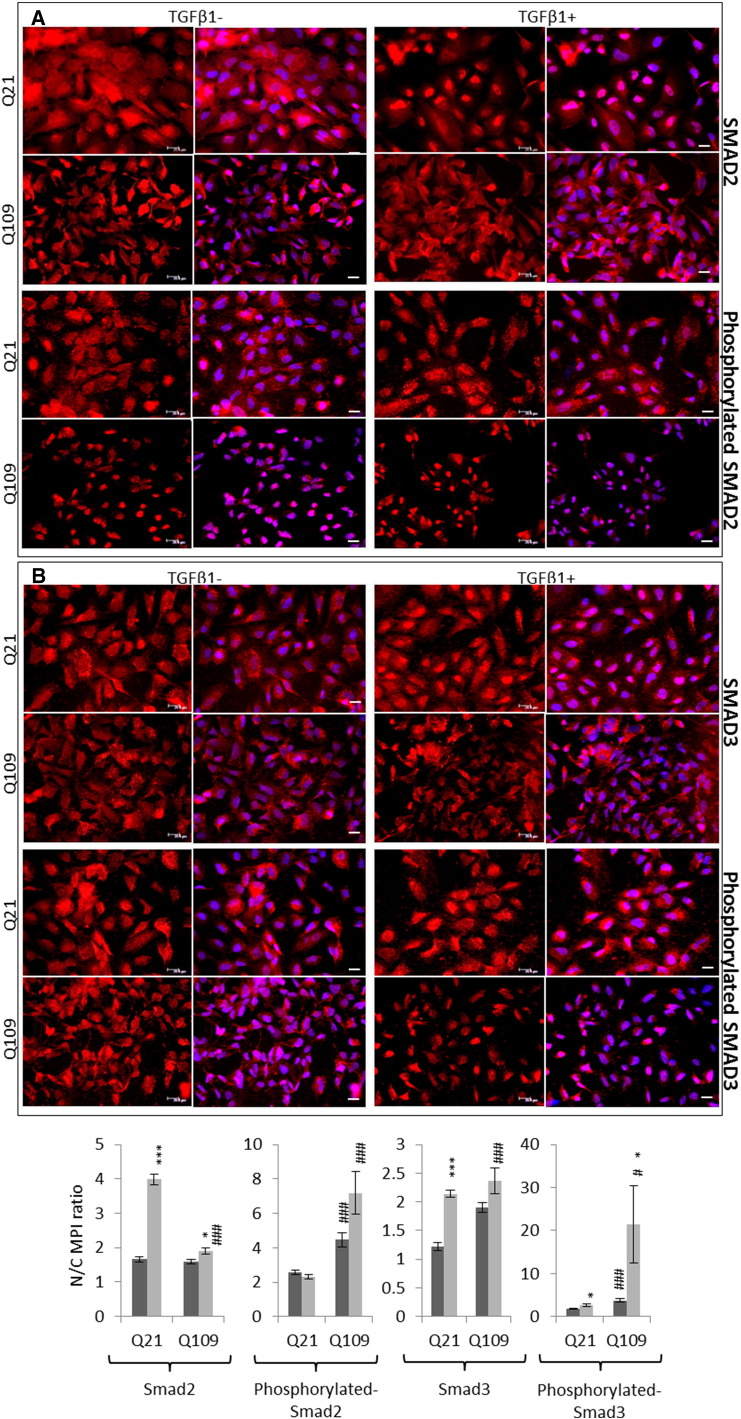
Total and phosphorylated SMAD2 and SMAD3 localisation in human NPC lines. Fixed Q21 and Q109 iPSCs stimulated with either 0 or 100 ng/ml TGFβ for 30 min, stained with antibodies against ***A***. SMAD2 and phosphorylated SMAD2, and ***B.*** SMAD3 and phosphorylated SMAD3, and nuclei counterstained with DAPI (blue). Scale bar = 20 μM. Figures are representative of multiple images. *N* = 3. **C**. Quantification of the nuclear/cytoplasmic mean pixel intensity (N/C MPI) ratio of the experiments presented in ***A*** and ***B***, based on an average of 90 cells per condition, taken from 6 different frames across 3 experiments (see Section 2, Methods). Asterisks denote a significant difference from 0 ng/ml TGFβ, hashes indicate a significant difference from Q21 cells. */# *p* < 0.05, **/## *p* < 0.01, ***/### *p* < 0.001.

The regulation of gene expression by SMAD transcription factors following activation of the TGFβ pathway involves cytoplasmic-nuclear shuttling where activated phospho-SMAD/co-SMAD complexes form transcriptional complexes and recruit DNA binding partners [7]. The subcellular localisation of both total and phosphorylated Smads 2 and 3 were therefore assayed by immunofluorescence. Typically, treatment with TGFβ1 initiated increased nuclear localisation of total and phosphorylated Smads 2 and 3 in all cell lines (Fig. 3, Fig. 4, Fig. 5). However, the presence of mHTT appeared to alter the extent of the nuclear accumulation of the Smads at both baseline, and following TGFβ1 stimulation.

There was little Smad2 accumulation in cell nuclei at baseline in *StHdh*^*Q7/7*^ and Q21 cells (Figs. 3; 5), however following TGFβ1 stimulation its localization was almost exclusively nuclear, which is also apparent in the nuclear/cytoplasmic (N/C) mean pixel intensity (MPI) ratios calculated for these cells (Figs. 3C; 5C). Both *StHdh*^*Q7/111*^ and *StHdh*^*Q111/111*^ cell lines had increased nuclear Smad2 at baseline compared with *StHdh*^*Q7/7*^ cells. Although there was movement into cell nuclei following TGFβ1 stimulation, the accumulation of Smad2 was not as exclusively nuclear as in *StHdh*^*Q7/7*^ cells, but remained in the area immediately surrounding the nucleus (Fig. 3). In contrast, despite several individual cells exhibiting high levels of nuclear SMAD2 in Q109 cells, there was very little effect of TGFβ1 on overall SMAD2 subcellular localisation. Phosphorylated Smad2 was primarily cytoplasmic at baseline in *StHdh*^*Q7/7*^ and Q21 cells, although its nuclear presence increased with *mHtt* gene dosage. There was movement of phosphorylated Smad2 into the nucleus in all three *StHdh*^*Q111*^ lines and both NPC lines following TGFβ1 stimulation (Figs. 3; 5).

At baseline, the localisation of Smad3 was similar between *StHdh*^*Q7/7*^, *StHdh*^*Q7/111*^ and *StHdh*^*Q111/111*^ cells (Fig. 4), but following TGFβ1 stimulation, nuclear accumulation appeared higher in *StHdh*^*Q111/111*^ cells compared to both *StHdh*^*Q7/111*^ and *StHdh*^*Q7/7*^ lines. Similarly, SMAD3 localisation was increasingly nuclear in Q21 and Q109 cells following TGFβ1 stimulation (Fig. 5), although to a lesser extent in Q109 NPCs.

Nuclear over-accumulation of phosphorylated Smad3 was less apparent in *StHdh*^*Q111/111*^ cells following TGFβ1 stimulation (Fig. 4), and there was high variability between cells as some individual cells failed to accumulate nuclear phosphorylated Smad3 to the same extent as other cells in the same frame and as in *StHdh*^*Q7/7*^ cells (Fig. 4). In both NPC lines, phosphorylated SMAD3 was initially more nuclear at baseline than total SMAD3, and while it increased in the nuclei of Q21 cells, this effect was less clear in Q109 cells; although this again appeared to be due to high variability between individual cells (Fig. 5).

### 3.5 SMAD phosphorylation is inhibited in cell models of HD

**Fig. 6:**
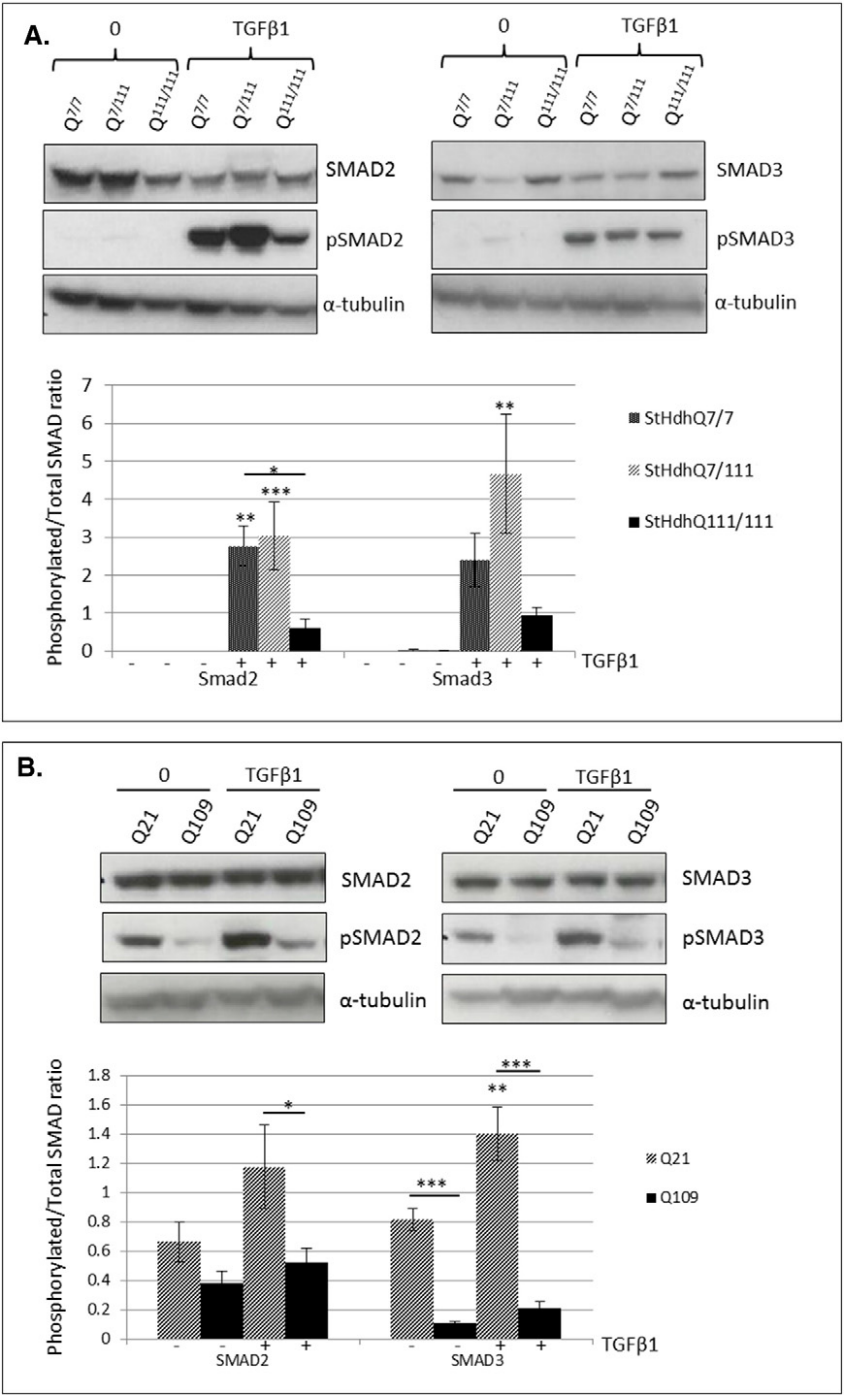
Western blot analysis of total and phosphorylated SMADs 2 and 3 in ***A.****StHdh*^*Q111*^ cell lines and ***B***. NSCs, prior to, and following 30 min incubation with 100 ng/ml TGFβ1, and representation of phosphorylated/total SMAD ratios from densitometry analysis of western blot images. All densitometry analyses of SMAD proteins are normalised to α-tubulin. * *p* < 0.05, ** *p* < 0.01, *** *p* < 0.001. Images representative of multiple experiments. *N* = 5.

SMAD protein expression and phosphorylation were determined in mouse *StHdh* and human iPSC-derived NPC lines by western blot. There was a reduction in total Smad2 detection in both *StHdh*^*Q7/7*^ (*p* < 0.01) and *StHdh*^*Q7/111*^ (*p* < 0.05) cells following TGFβ1 stimulation, but no effect in the *StHdh*^*Q111/111*^ line (Fig. 6a); the reason for this reduction is unclear, although may be a result of induction of the ubiquitin-proteasome system [17], or a technical artefact resulting from increased Smad2 phosphorylation and therefore potentially reduced specificity of the total Smad2 antibody. There was also no effect of genotype on the level of total SMAD2 detected in the human NPCs (Fig. 6b). For all three *StHdh* lines, there was a clear phosphorylation response in Smad2 following a 30 min treatment with TGFβ1 (Fig. 6A), which was similar in extent in both *StHdh*^*Q7/7*^ and *StHdh*^*Q7/111*^ cell lines (both *p* < 0.001), but did not reach significance in *StHdh*^*Q111/111*^ cells. As a result, following TGFβ1 stimulation, the level of phosphorylated Smad2 in *StHdh*^*Q111/111*^ cells was significantly lower compared to both *StHdh*^*Q7/7*^ (*p* < 0.05) and *StHdh*^*Q7/111*^ (*p* < 0.001) cells. The same pattern is present in the phosphorylated/total Smad2 ratio in human cells (Fig. 6b). Similar to *StHdh* lines, SMAD2 did exhibit some phosphorylation following TGFβ1 stimulation, although this response was not as pronounced as in the immortalised mouse cell line. However, there was a trend towards lower levels of phosphorylated SMAD2 in Q109 cells, and a significantly suppressed response following TGFβ1 stimulation (*p* < 0.05). The same pattern of effect was also apparent in the phosphorylated/total SMAD2 ratio (Fig. 6b).

There was no apparent effect of either genotype or TGFβ1 stimulation on the level of total Smad3 protein expression in either the mouse *StHdh* cells or in the human NPC lines (Fig. 6). Similar to the effect on Smad2, there was a substantial effect of TGFβ1 stimulation on the phosphorylation of all three cell lines (*StHdh*^*Q7/7*^ *p* < 0.01, *StHdh*^*Q7/111*^ *p* < 0.001, *StHdh*^*Q111/111*^ *p* < 0.05; Fig. 6). However, there was only a modest phosphorylation response in the human NPC lines, and significantly less phosphorylated SMAD3 in Q109 cells at both baseline (*p* < 0.001) and following TGFβ1 stimulation compared to Q21 NPCs (Fig. 6). Calculation of the phosphorylated/total Smad3 ratio demonstrates that there is a an increased phosphorylation response in *StHdh*^*Q7/111*^ cells compared to both *StHdh*^*Q7/7*^ (*p* < 0.01) and *StHdh*^*Q111/111*^ (*p* < 0.05) lines, and that the same phosphorylation response is suppressed in *StHdh*^*Q111/111*^ cells (*p* < 0.05; Fig. 6. Similar to the *StHdh*^*Q111/111*^ cells, the level of phosphorylated SMAD3 in Q109 cells remains significantly lower at both baseline and following TGFβ1 stimulation compared to Q21 cells following calculation of the phosphorylated/total SMAD3 ratio (both *p* < 0.001; Fig. 6).

### 3.6 SMAD3 associates with the *HTT* promoter

**Supplementary Fig. 4:**
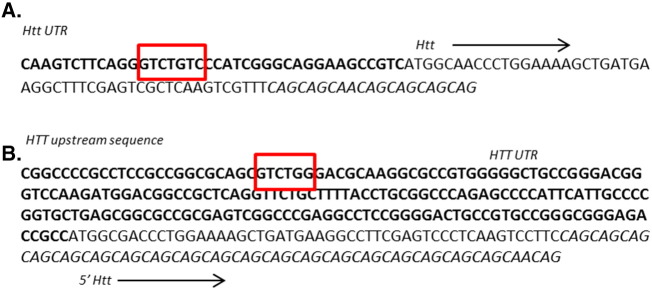
SMAD binding elements in HTT UTR. **A.** Mouse and **B.** Human 5′ *HTT* and upstream untranslated region (UTR; in Bold). SMAD binding element (GTCTG) is highlighted with a red box. CAG repeat is in italics.

A Smad binding element (SBE) consensus sequence was identified in the upstream promoter region of both mouse *Htt* and human *HTT* (Supplementary Fig. 4). Chromatin immunoprecipitation (ChIP) was therefore carried out in order to determine whether Smad TFs bind to the *Htt* promoter in *StHdh*^*Q111*^ cells.

**Fig. 7:**
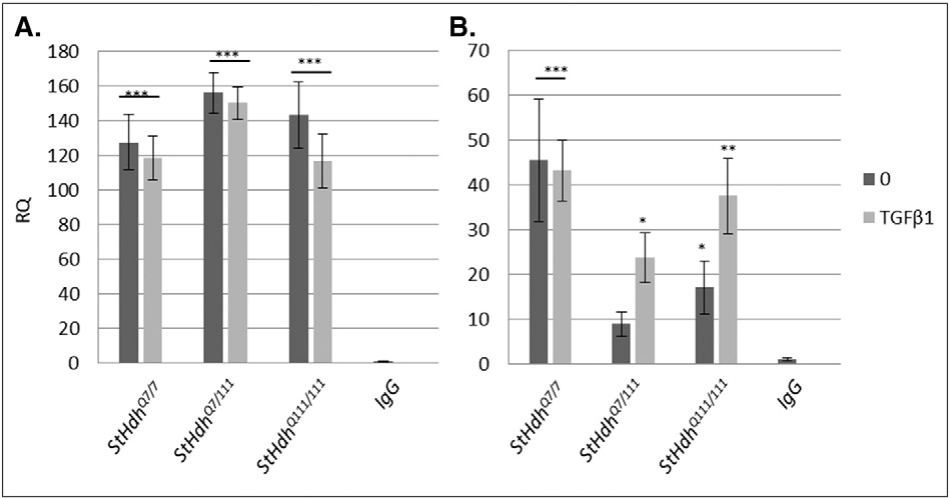
qPCR data demonstrating the enrichment of *Htt* DNA from ChIP using **A.** Smad3 and **B**. phosphorylated Smad3 antibodies, analysed using the fold-enrichment method (see Section 2, Methods). Asterisks denote a significant difference in precipitated *Htt* using either total or phosphorylated Smad3 antibodies compared to the IgG control. * *p* < 0.05, ** *p* < 0.01, *** *p* < 0.001. *N* = 3.

ChIP was carried out using antibodies against both Smad3 and phosphorylated Smad3, as we observed a consistent dysregulation of its phosphorylation and subcellular localisation in both *StHdh* and iPSC-derived NPC lines. *Htt* promoter DNA was immunoprecipitated with the total Smad3 antibody, but there were no differences between genotypes (Fig. 7). However, the antibody against phosphorylated Smad3 pulled down substantial levels of *Htt* promoter sequence in both baseline and TGFβ1 stimulated *StHdh*^*Q7/7*^ cells (Fig. 7). In *StHdh*^*Q7/111*^ and *StHdh*^*Q111/111*^ cell lines, significantly less *Htt* DNA was immunoprecipitated; in *StHdh*^*Q111/111*^ cells at baseline, the amount of *Htt* detected in the precipitated DNA was significantly higher than in the IgG control, although not in *StHdh*^*Q7/111*^ cells. TGFβ1 stimulation increased the level of immunoprecipitated *Htt* in *StHdh*^*Q7/111*^ and *StHdh*^*Q111/111*^ cells (Fig. 7). We therefore examined whether *HTT* expression was regulated by SMAD3 activation by using a specific inhibitor of Smad 2 and 3 phosphorylation; SB525334.

**Supplementary Fig. 5:**
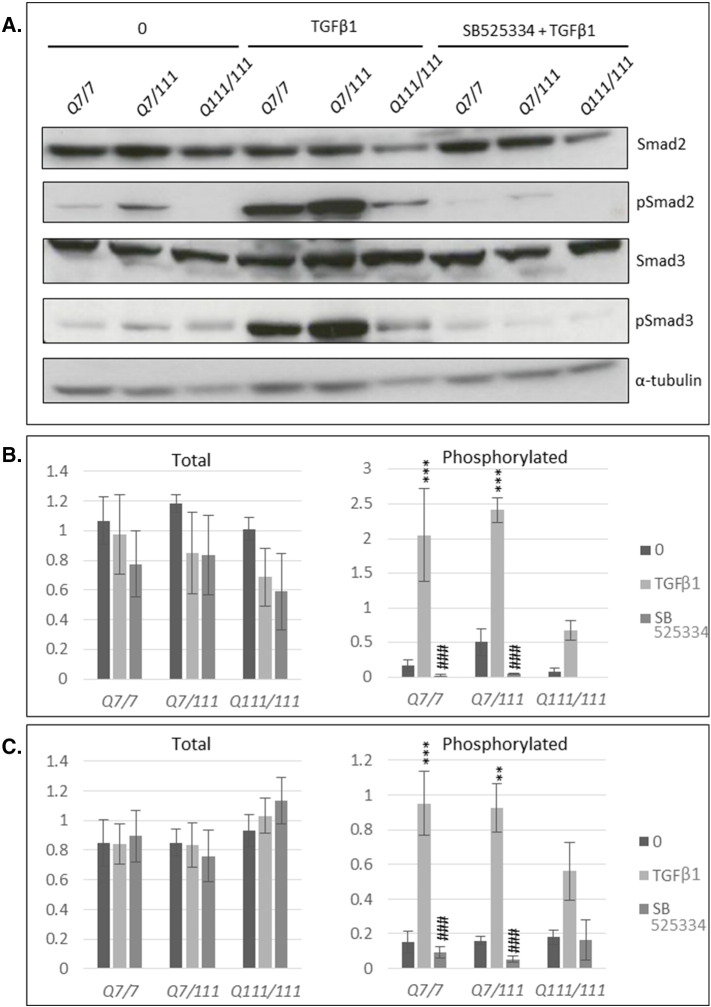
Validation of SB525334 inhibition by western blot in *StHdh* cells. ***A.*** Detection of total and phosphorylated Smads 2 and 3 in *StHdh*^*Q111*^ cells by western blot at baseline (0), or following 30 min 100 ng/ml TGFβ stimulation, either with or without prior treatment with SB525334***. B—C***. Densitometry analysis of Smad2 blots (***B***) and Smad3 blots (***C***) in *A*, normalised to α-tubulin. Asterisks indicate a significant difference from baseline, and hashes indicate a significant difference from TGFβ1 stimulation. *** *p* < 0.001. Images representative of multiple blots *N* = 3.

**Supplementary Fig. 6:**
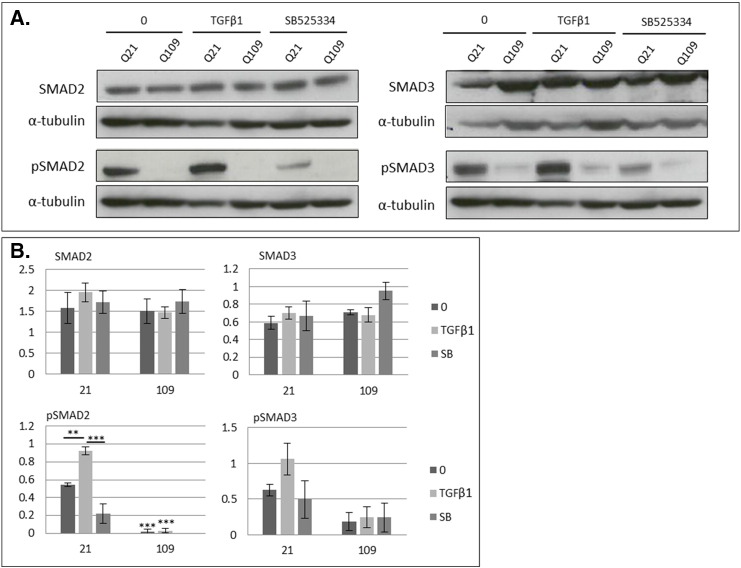
Validation of SB525334 inhibition by western blot in human NPCs*.****A****.* Detection of total and phosphorylated SMADs 2 and 3 in Q21 and Q109 NSCs by western blot at baseline (0), or following 30 min 100 ng/ml TGFβ1 stimulation, either with or without prior treatment with SB525334. ***B***. Densitometry analysis of blots in *A*, normalised to α-tubulin. ** *p* < 0.01, *** *p* < 0.001. Images representative of multiple blots, *N* = 3

**Supplementary Fig. 7:**
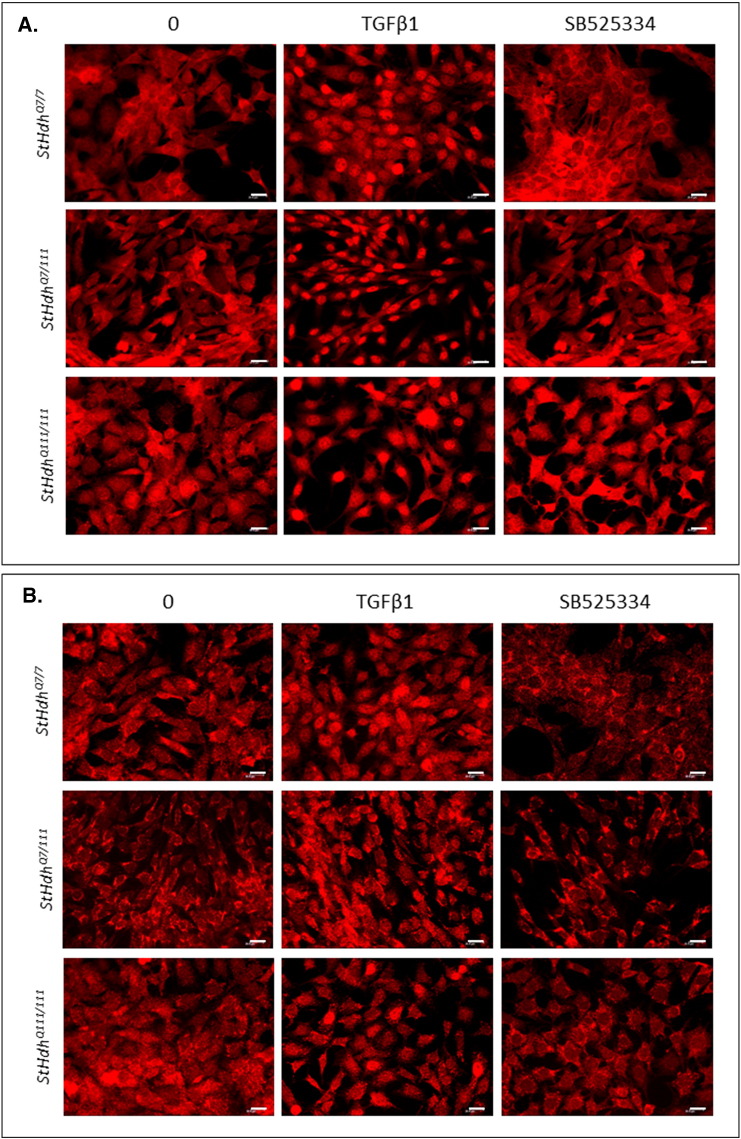
Validation of SB525334 inhibition by immunofluorescence in *StHdh* cells. Fixed *StHdh*^*Q111*^ cells at baseline, and following treatment with 100 ng/ml TGFβ1, either with or without prior treatment with SB525334, and stained with antibodies against ***A.*** Smad2 and ***B***. Smad3. Scale bar = 20 μM. Figures representative of multiple images, *N* = 3

**Supplementary Fig. 8:**
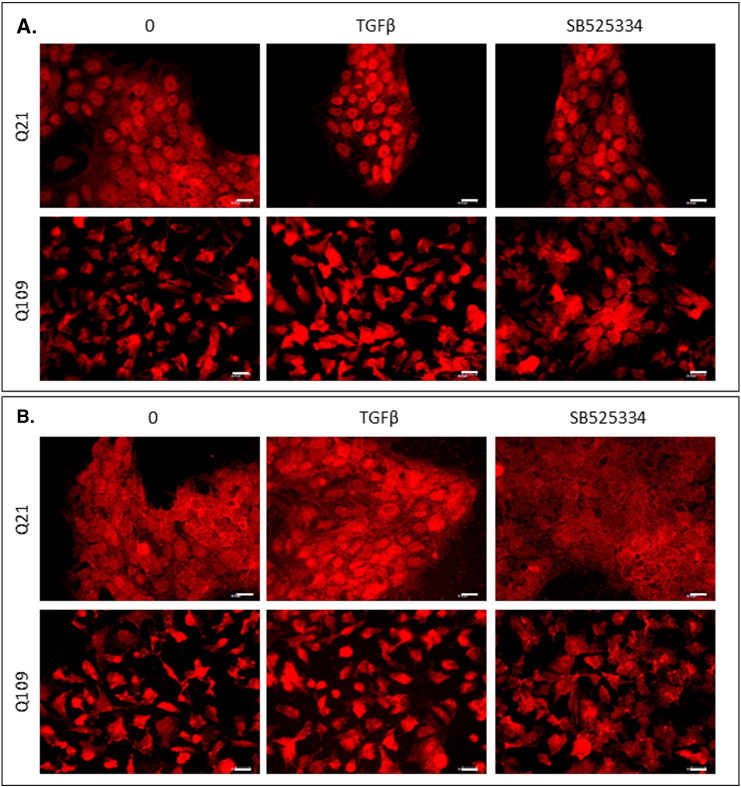
Validation of SB525334 inhibition by immunofluorescence in human NPCs*.* Fixed Q21 and Q109 NSCs at baseline, and following treatment with 100 ng/ml TGFβ1, either with or without prior treatment with SB525334, and stained with antibodies against ***A.*** SMAD2 and ***B.*** SMAD3. Scale bar = 20 μM. Figures representative of multiple images, *N* = 3

**Fig. 8:**
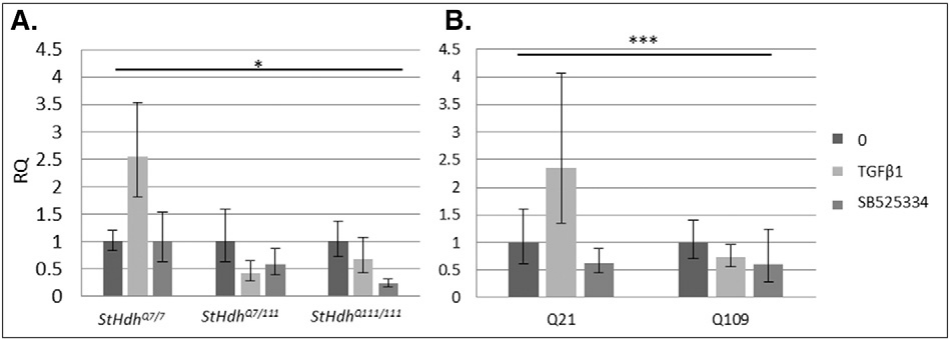
*HTT* expression following SMAD inhibition. RQ values from qRTPCR for the expression of *HTT* in ***A.****StHdh*^*Q111*^ cells and ***B.*** NPCs following TGFβ1 stimulation and inhibition with SB525334. There was a significant effect of genotype on *HTT* expression in *StHdh*^*Q111*^ cells (***A***; F8,38 = 2.56, *p* < 0.05) and in NPCs (***B***; *F*1, 25 = 22.9333, *p* < 0.001). *N* = 6.

Validation of the effects of SB525334 on Smad activation was carried out in both *StHdh* and NPC lines using western blot and immunofluorescence (Supplementary Fig. 5, Supplementary Fig. 6, Supplementary Fig. 7, Supplementary Fig. 8). There was a trend towards a TGFβ1-induced increase in *Htt* expression in *StHdh*^*Q7/7*^ cells, which was blocked by inhibition with SB525334 (Fig. 8). In contrast, while SB525334 inhibition of Smads 2 and 3 reduced *Htt* expression in *StHdh*^*Q7/111*^ and *StHdh*^*Q111/111*^ cells, the effect was less marked due to a lack of effect of TGFβ1 alone.

The expression of *HTT* in Q21 and Q109 cells following SB525334 treatment was also determined by qRTPCR (Fig. 8). *HTT* expression was consistently higher in Q21 compared with the Q109 cells and TGFβ1 elicited a trend towards increased *HTT* expression in Q21 cells, which was prevented by treatment with SB525334. There was no observable effect of SB525334 on the expression of *HTT* in Q109 cells, although this is likely due to the absence of an initial effect of TGFβ1.

## 4 Discussion

By investigating differential gene expression between *StHdh*^*Q7/7*^ and *StHdh*^*Q111/111*^ cell lines using microarray analysis, we identified TGFβ signalling as a significantly altered signalling pathway, despite using EGF as a stimulus. This is not surprising, as substantial crosstalk between EGF and TGFβ pathways has previously been identified [18], [19], [20], [21]; for example, while *SMAD7* is an inhibitor of SMAD signalling by competitive interaction with the TGFβ type 1 receptor [22] and by interfering with SMAD-SMAD interactions [7], it is also responsive to EGF stimulation in order to act as a negative regulator of this pathway [23]. Another significantly differentially expressed gene identified from our microarray analysis*, Spry2*, is also known to mediate the crosstalk between these two pathways; its activation is increased by stimulation of the EGF receptor (EGFR), where it then propagates the signal by preventing degradation of the EGFR. TGFβ suppresses the transcription of *Spry2*, degrading its protein and suppressing EGFR activation [24]. Enhancement of the TGFβ signalling pathway would therefore be consistent with a suppressed response to EGF, which we observe in *StHdh*^*Q111/111*^ cells both in these microarray data, and in our previously published work [16].

The genes that were identified as having a stronger transcriptional response in *StHdh*^*Q111/111*^ cells encode a diverse range of functions, predominantly cellular survival and proliferation. However, the majority of genes on these lists are known to be responsive to TGFβ1 stimulation, and several have direct associations with SMAD transcription factors (TFs). For example, the HMGA2 protein regulates a feed forward mechanism of gene transcription via a direct interaction with SMADs 3 and 4 [25]. *Irf2bp2* encodes a muscle enriched TF [26] that has been found to be a TGFβ responsive gene [27], as are *Cdc42ep3* and *Gadd45g* [28], [29], [30].

Our identification of TGFβ signalling as a significantly altered pathway in a cell model of HD is consistent with a recent RNA-seq analysis of HD patient iPSC-derived NPCs, where TGFβ signalling was one of the most significantly dysregulated pathways [11], and targets of SMAD3 were over-represented within genes differentially expressed between HD-NPCs and their isogenic controls [11].

In order to further characterise the TGFβ signalling pathway, we focused on the regulation of SMAD TFs in the mouse *StHdh*^*Q111*^ model and in human iPSC-derived NPCs. The most consistent observation was for suppressed expression of the inhibitory *SMAD*, *SMAD7,* in response to TGFβ1 stimulation in *StHdh*^*Q111/111*^ and in Q109 NPCs, which would be suggestive of reduced inhibition of TGFβ signalling. However, as the remaining expression data and protein characterisation data do not indicate a substantial increase in TGFβ signalling activity, the lack of *SMAD7* responsiveness may be a compensatory mechanism in these cells attempting to enhance kinase signalling and mediate transcriptional regulation.

Immunofluorescence analysis of *StHdh*^*Q7/7*^ cells and Q21 NPCs demonstrates that stimulation with TGFβ1 is activating SMAD TFs by inducing phosphorylation of SMADs 2 and 3 and their nuclear localisation, which is required for SMAD-regulated transcriptional activity. This translocation is altered in cells containing a CAG expansion. In particular, Smad2 exhibits increased nuclear localisation in *StHdh*^*Q7/111*^ and *StHdh*^*Q111/111*^ cells, and phosphorylated SMAD2 is predominantly nuclear in Q109 NPCs prior to TGFβ1 stimulation. This is consistent with the increased SMAD2 phosphorylation identified in other HD patient-derived NPCs [11]. Altered localisation and phosphorylation of SMAD3 is also observed in both cell lines, although it is more consistent with a generalised dysregulation and disordered response to TGFβ1, than nuclear accumulation in particular. As we do not observe an augmented phosphorylation response in CAG-repeat expansion cell lines in response to TGFβ1 by western blot, it is possible that the increased nuclear localisation of these factors, combined with suppressed *SMAD7* expression and inhibition, may be a compensatory mechanism to retain higher TGFβ pathway activity in an otherwise detrimental environment.

An attempt to increase TGFβ signalling may be indicative of a protective response in these cells; increasing TGFβ1 in cellular growth media was found to reduce the activity of caspases 3 and 7 [11], and increased expression of TGFβ1 has been associated with neuroprotection, astrogliosis and alleviation of neuroinflammation [31], [32], whereas its knockdown promoted neuronal loss in rats following traumatic brain injury [32]. However, as microglia and astrocytes both express TGFβ1 and are inextricably involved in these neuroprotective processes [31], [32], [33], [34], the culture of neuronal-like cells in isolation from these other neural cell types will not be fully sufficient for the elucidation of the potential neuroprotective effects of increased TGFβ1 in models of HD.

In addition to characterising the altered regulation of SMAD TFs in *StHdh*^*Q111*^ cells and iPSC-derived NPCs, we demonstrate that a SMAD binding element is present within the *HTT* promoter region, and SMAD3 can bind directly to the *Htt* promoter in *StHdh*^*Q111*^ cells, suggesting that SMAD3 activity may directly regulate the expression of *Htt*. This was supported by the increased expression of *HTT* in both *StHdh*^*Q7/7*^ and Q21 cells following TGFβ1 stimulation, and the suppression of this effect when SMAD activation was inhibited with the compound SB525334. The increased expression of *HTT* following TGFβ1 stimulation was not present in cells with an expanded CAG repeat, which is consistent with the reduced affinity of phosphorylated SMAD3 for the *Htt* promoter in *StHdh*^*Q7/111*^ and *StHdh*^*Q111/111*^ cells. As TGFβ is required for neurogenesis [35], as well as for midbrain dopaminergic neuronal development [36], and HTT has also been implicated in the regulation of neurogenesis [37], [38] and is essential for embryonic development [39], it is possible that TGFβ signalling may be directly regulating *HTT* expression in order for these processes to occur.

## 5 Conclusions

• We implicate dysregulation of the TGFβ signalling pathway in mouse and human cell models of HD, consistent with recent RNA-seq data from hiPSC-derived NPCs [11].
• Characterisation of the downstream SMADs within this pathway uncovered widespread dysregulation, which may indicate a compensatory, neuroprotective response to the CAG expansion.
• Finally, we demonstrate that SMAD3 binds to the *HTT* promoter, and that *HTT* expression can be regulated by SMAD3 activation in the absence of the CAG expansion.
• This may have implications for neurogenesis and striatal development. TGFβ signalling is therefore a possible target for disease modification and could prove to be a useful biomarker for disease progression.

The following are the supplementary data related to this article.

Supplementary tables Image 1

## Figures and Tables

**Table 1:** Clustered functional annotations for genes differentially expressed between baseline and following EGF stimulation. Significant DAVID functional annotations following our clustering mechanism for *A*. genes differentially expressed in *StHdh*^*Q7/7*^ and *StHdh*^*Q111/111*^ cells between baseline and following EGF stimulation, and *B*. genes with a significant EGF x Genotype interaction.

A				
Node	*StHdh*^*Q7/7*^		*StHdh*^*Q111/111*^	
	*p*-value	No. genes	*p*-value	No. genes
PIRSF001719:FOS TRANSFORMING PROTEIN			9.52 × 10^− 4^	3
GO:0051789 ~ RESPONSE TO PROTEIN STIMULUS			1.52 × 10^− 3^	6
GO:0048589 ~ DEVELOPMENTAL GROWTH			2.81 × 10^− 3^	6
GO:0003002 ~ REGIONALIZATION			2.28 × 10^− 4^	10
DEVELOPMENTAL PROTEIN			3.47 × 10^− 3^	16
GO:0006355 ~ REGULATION OF TRANSCRIPTION, DNA-DEPENDENT	8.13 × 10^− 12^	45	9.16 × 10^− 13^	45
GO:0048514 ~ BLOOD VESSEL MORPHOGENESIS	1.35 × 10^− 6^	13		
IPR013087:ZINC FINGER, C2H2-TYPE/INTEGRASE, DNA-BINDING	6.31 × 10^− 5^	15		
GO:0045859 ~ REGULATION OF PROTEIN KINASE ACTIVITY	1.50 × 10^− 4^	10		
GO:0048729 ~ TISSUE MORPHOGENESIS	2.13 × 10^− 4^	11		
GO:0051094 ~ POSITIVE REGULATION OF DEVELOPMENTAL PROCESS	4.65 × 10^− 4^	10	2.28 × 10^− 4^	10
MMU04010:MAPK SIGNALLING PATHWAY	4.88 × 10^− 4^	11	5.26 × 10^− 5^	11
GO:0044092 ~ NEGATIVE REGULATION OF MOLECULAR FUNCTION	2.86 × 10^− 3^	7		
GO:0009611 ~ RESPONSE TO WOUNDING	3.14 × 10^− 3^	11		
GO:0048511 ~ RHYTHMIC PROCESS	3.71 × 10^− 3^	6	3.47 × 10^− 4^	7


B		
Node	*p*-value	No. genes
PHOSPHOPROTEIN	8.01 × 10^− 14^	228
GO:0042127 ~ REGULATION OF CELL PROLIFERATION	1.08 × 10^− 11^	44
TRANSCRIPTION REGULATION	5.48 × 10^− 10^	75
GO:0048598 ~ EMBRYONIC MORPHOGENESIS	2.43 × 10^− 8^	30
GO:0040007 ~ GROWTH	1.55 × 10^− 6^	19
GO:0007507 ~ HEART DEVELOPMENT	3.10 × 10^− 6^	20
MMU04010:MAPK SIGNALLING PATHWAY	3.42 × 10^− 6^	22
MMU04350:TGF-BETA SIGNALLING PATHWAY	1.21 × 10^− 5^	12
GO:0045596 ~ NEGATIVE REGULATION OF CELL DIFFERENTIATION	1.26 × 10^− 5^	17
GO:0051789 ~ RESPONSE TO PROTEIN STIMULUS	5.81 × 10^− 5^	11
GO:0010941 ~ REGULATION OF CELL DEATH	7.28 × 10^− 5^	31
GO:0051094 ~ POSITIVE REGULATION OF DEVELOPMENTAL PROCESS	9.11 × 10^− 5^	17
GO:0048608 ~ REPRODUCTIVE STRUCTURE DEVELOPMENT	9.60 × 10^− 5^	13
GO:0008201 ~ HEPARIN BINDING	1.75 × 10^− 4^	10
GO:0022405 ~ HAIR CYCLE PROCESS	2.20 × 10^− 4^	8
GO:0043583 ~ EAR DEVELOPMENT	2.61 × 10^− 4^	11
METAL-BINDING	3.03 × 10^− 4^	96
MMU05210:COLORECTAL CANCER	3.35 × 10^− 4^	10
GO:0007242 ~ INTRACELLULAR SIGNALLING CASCADE	3.49 × 10^− 4^	41
GROWTH FACTOR	6.52 × 10^− 4^	11
GO:0007178 ~ TRANSMEMBRANE RECEPTOR PROTEIN SERINE/THREONINE KINASE SIGNALLING PATHWAY	6.96 × 10^− 4^	10
GO:0006928 ~ CELL MOTION	8.90 × 10^− 4^	21
DEVELOPMENTAL PROTEIN	9.92 × 10^− 4^	34
GO:0009968 ~ NEGATIVE REGULATION OF SIGNAL TRANSDUCTION	1.18 × 10^− 3^	13
GO:0048145 ~ REGULATION OF FIBROBLAST PROLIFERATION	1.33 × 10^− 3^	5
GO:0001890 ~ PLACENTA DEVELOPMENT	1.43 × 10^− 3^	9
MMU05222:SMALL CELL LUNG CANCER	1.46 × 10^− 3^	9
GO:0048008 ~ PLATELET-DERIVED GROWTH FACTOR RECEPTOR SIGNALLING PATHWAY	2.29 × 10^− 3^	5
GO:0030155 ~ REGULATION OF CELL ADHESION	2.35 × 10^− 3^	9
ALTERNATIVE SPLICING	2.63 × 10^− 3^	130
GO:0017017 ~ MAP KINASE TYROSINE/SERINE/THREONINE PHOSPHATASE ACTIVITY	2.64 × 10^− 3^	4
CLEAVAGE ON PAIR OF BASIC RESIDUES	3.25 × 10^− 3^	14
GO:0045165 ~ CELL FATE COMMITMENT	3.72 × 10^− 3^	11
GO:0008285 ~ NEGATIVE REGULATION OF CELL PROLIFERATION	4.05 × 10^− 3^	14
GO:0032869 ~ CELLULAR RESPONSE TO INSULIN STIMULUS	4.10 × 10^− 3^	6
GO:0001570 ~ VASCULOGENESIS	4.10 × 10^− 3^	6
CYCLIN	4.55 × 10^− 3^	6
GO:0021915 ~ NEURAL TUBE DEVELOPMENT	4.56 × 10^− 3^	8
APOPTOSIS	4.66 × 10^− 3^	17
GO:0051056 ~ REGULATION OF SMALL GTPASE MEDIATED SIGNAL TRANSDUCTION	4.71 × 10^− 3^	14
GO:0045765 ~ REGULATION OF ANGIOGENESIS	5.00 × 10^− 3^	6

**Table 2:** Fold change gene expression between *StHdh*^*Q7/7*^ and *StHdh*^*Q111/111*^ cells for TGFβ pathway-related genes following microarray analysis.

Gene ID	Gene name	*StHdh*^*Q111/111*^ vs *StHdh*^*Q7/7*^ fold change	*p*-value
*Smurf2*	Smad specific E3 ubiquitin protein ligase 2	1.74	2.1 × 10^− 13^
*Tgfβ1*	Transforming growth factor beta 1	− 1.5	2.4 × 10^− 14^
*Tgfβr1*	TGF-β receptor 1	− 1.2	1.6 × 10^− 5^
*Tgfβ2*	Transforming growth factor beta 2	1.5	1.2 × 10^− 14^
*Tgfβr2*	TGF-β receptor 2	3	5.2 × 10^− 33^
*Tgfβ3*	Transforming growth factor beta 3	3	2.0 × 10^− 25^
*Tgfβr3*	TGF-β receptor 3	6	2.1 × 10^− 27^
*Tgfβi*	TGF-β induced protein	11.4	2.1 × 10^− 30^
*Smad1*	Mothers against decapentaplegic homolog 1	− 1	4.5 × 10^− 1^
*Smad2*	Mothers against decapentaplegic homolog 2	− 1.2	7.8 × 10^− 9^
*Smad3*	Mothers against decapentaplegic homolog 3	1.27	1.20 × 10^− 12^
*Smad4*	Mothers against decapentaplegic homolog 4	− 1.03537	3.9 × 10^− 2^
*Smad5*	Mothers against decapentaplegic homolog 5	− 1.16	1.7 × 10^− 9^
*Smad6*	Mothers against decapentaplegic homolog 6	1.6	3.27 × 10^− 15^
*Smad7*	Mothers against decapentaplegic homolog 7	− 1.32277	1.69 × 10^− 9^
