# Supplementary material for: SMAD transcription factors are altered in cell models of HD and regulate HTT expression
Source: Cell Signal. 2017 Feb;31:1–14. doi: 10.1016/j.cellsig.2016.12.005 (PMC5310119; doi:10.1016/j.cellsig.2016.12.005)
Supplement: Supplementary file 1 — Supplementary tables [file mmc1.doc]

**Supplementary Table 1**. Parameters for creating gene lists for pathway analyses. FDR = false discovery rate

| **Comparison** | **Fold Change** | **FDR** |
| --- | --- | --- |
| *StHdh^Q7/7^* 0 hours vs. *StHdh^Q111/111^* 0 hours | >2 - <-2 | 0.05 |
| *StHdh^Q7/7^* 0 hours vs*. StHdh^Q7/7^* 2 hours | >1.5-<-1.5 | 0.05 |
| *StHdh^Q111/111^* 0 hours vs*. StHdh^Q111/111^* 2 hours | >1.5-<-1.5 | 0.05 |
| Interaction | >2 - <-2 | 0.05 |

**Supplementary Table 2. Details of the primary and secondary antibodies used for immunofluorescence and western blotting during this project.**

| Antibody | IF dilution | WB dilution | Supplier |
| --- | --- | --- | --- |
| **Primaries** | | | |
| SMAD1 | 1:200 | 1:1000 | NEB |
| phosphoSMAD1 (ser465) | 1:100 | 1:1000 | Abcam |
| SMAD2 | 1:100 | 1:1000 | NEB |
| PhosphoSMAD2 (ser465/467) | / | 1:1000 | NEB |
| PhosphoSMAD2 (ser465/467) | 1:500 | / | Millipore |
| SMAD3 | 1:100 | 1:1000 | NEB |
| PhosphoSMAD3 (ser423/425) | / | 1:1000 | NEB |
| PhosphoSMAD3 (ser423/425) | 1:100 | / | Abcam |
| SMAD4 | / | 1:1000 | Abcam |
| SMAD4 | 1:50 | / | Abgent |
| SMAD5 | 1:1000 | 1:1000 | Abcam |
| PhosphoSMAD5 (ser463/465) | 1:100 | 1:1000 | Abcam |
| SMAD6 | 1:125 | 1:1000 | Antibodies Online |
| SMAD7 | 1:160 | 1:800 | Abcam |
| α-tubulin | / | 1:5000 | Abcam |
| Nestin | 1:500 | / | Abcam |
| Foxp2 | 1:500 | / | Abcam |
| Sox2 | 1:500 | / | Abcam |
| Pax6 | 1:100 | / | Abcam |
| Secondaries | | | |
| Goat-anti-rabbit 568 | 1:100 | / | LifeTech |
| Donkey-anti-mouse 488 | 1:100 | / | LifeTech |
| Anti-Rabbit HRP | / | 1:15,000 | Vector Labs |
| Anti-Mouse HRP | / | 1:15,000 | Vector Labs |

**Supplementary Table 3. Validation of EGF stimulation-regulated genes by qRTPCR.** Microarray gene expression fold change data for *StHdh^Q111/111^* and *StHdh^Q7/7^* cells following 2 hours 100ng/ml EGF stimulation are given with their associated p-values. In comparison is the ratio of the qRTPCR RQ value following EGF stimulation compared with the corresponding RQ value at baseline, and the associated p-value. qRTPCR data was analysed using a two-way ANOVA followed by post-hoc Tukey tests. A ‘ü‘ indicates that gene expression was fully validated, non-validated genes are noted with a ‘û,’ and genes that showed the same pattern of expression as the microarray data but did not reach significance following qRTPCR analysis are marked with ‘≈.’ n=5.

|  |  | Microarray | | qRTPCR | | Validated? |
| --- | --- | --- | --- | --- | --- | --- |
| Gene | Genotype | Fold change | P-value | RQ value ratio | P-value |  |
| *Id3* | *StHdh^Q7/7^* | -1.75 | 1.8 x -10^11^ | 0.3 | 0.001 | ✓ |
|  | *StHdh^Q111/111^* | -2.18 | 3.6 x -10^14^ | 0.21 | 0.001 |  |
| *Atf3* | *StHdh^Q7/7^* | 1.56 | 2.2 x -10^8^ | 1.86 | n.s | ✓ |
|  | *StHdh^Q111/111^* | 1.98 | 1.0 x -10^11^ | 3.26 | 0.058 |  |
| *Egr1* | *StHdh^Q7/7^* | 1.54 | 9.2 x -10^13^ | 1.37 | n.s | ≈ |
|  | *StHdh^Q111/111^* | 1.56 | 1.2 x -10^12^ | 1.25 | n.s |  |
| *Fosl1* | *StHdh^Q7/7^* | 2.23 | 1.6 x -10^22^ | 3.36 | 0.002 | ✓ |
|  | *StHdh^Q111/111^* | 2.54 | 1.2 x -10^23^ | 3.37 | 0.001 |  |
| *Etv5* | *StHdh^Q7/7^* | 1.52 | 3.7 x -10^11^ | 0.69 | n.s | χ |
|  | *StHdh^Q111/111^* | 1.74 | 2.4 x -10^13^ | 1.34 | n.s |  |
| *Gadd45g* | *StHdh^Q7/7^* | -1.24 | 1.9 x -10^5^ | 1.14 | n.s | ✓ |
|  | *StHdh^Q111/111^* | -1.89 | 1.7 x -10^14^ | 0.65 | 0.006 |  |
| *Ngf* | *StHdh^Q7/7^* | 1.17 | 1.2 x -10^5^ | 0.74 | n.s | ≈ |
|  | *StHdh^Q111/111^* | 2.01 | 3.7 x -10^18^ | 1.26 | n.s |  |
| *Rasa1* | *StHdh^Q7/7^* | 1.23 | 2.1 x -10^8^ | 1.22 | n.s | ✓ |
|  | *StHdh^Q111/111^* | 1.53 | 1.1 x -10^14^ | 1.96 | 0.09 |  |
| *Smad6* | *StHdh^Q7/7^* | -1.44 | 7.2 x -10^13^ | 0.3 | 0.005 | ✓ |
|  | *StHdh^Q111/111^* | -1.58 | 5.5 x -10^15^ | 0.4 | 0.021 |  |
| *Dusp5* | *StHdh^Q7/7^* | 2.87 | 1.2 x -10^17^ | 5.9 | 0.012 | ✓ |
|  | *StHdh^Q111/111^* | 1.78 | 7.9 x -10^12^ | 29 | 0.001 |  |

**Supplemental Table 4. Top 10 functional annotations following DAVID analysis of differentially expressed genes following EGF stimulation.** Comparison of the top 10 significant pathways identified by DAVID pathway analyses for genes differentially expressed following 2 hours 100ng/ml EGF stimulation in *StHdh^Q7/7^* and *StHdh^Q111/111^* cells, as well as for genes with a significant genotype x GF interaction. GO_BP = gene ontology term, biological processes. GO_MF = gene ontology term, molecular function. SP_PIR = protein information resource.

| Category | Term | p-value | | |
| --- | --- | --- | --- | --- |
|  |  | StHdh^Q7/7^ | StHdh^Q111/111^ | Interaction |
| GO_BP | Regulation of transcription from RNA polymerase II promoter | 6.10E-09 | 2.57E-09 | 8.10E-10 |
| SP_PIR | Transcription | 1.37E-08 | 1.94E-10 | 3.18E-09 |
| SP_PIR | Transcription Regulation | 7.04E-07 | 1.23E-07 | 5.48E-10 |
| GO_BP | Regulation of transcription, DNA dependent | 8.13E-12 | 9.16E-13 | - |
| GO_BP | Regulation of RNA metabolic process | 1.43E-11 | 1.59E-12 | - |
| GO_BP | Regulation of transcription | 1.28E-10 | 5.28E-12 | - |
| GO_MF | Transcription factor activity | 8.91E-08 | 1.33E-07 | - |
| GO_MF | Transcription regulator activity | 2.88E-08 | 6.85E-10 | - |
| SP_PIR | DNA-binding | 5.44E-08 | 3.58E-08 |  |
| GO_BP | Transcription | 2.48E-07 | - | - |
| INTERPRO | Zinc finger, C2H2-type/integrase, DNA-binding | - | 5.41E-08 | - |
| SP_PIR | Phosphoprotein | - | - | 8.01E-14 |
| GO_BP | Regulation of cell proliferation | - | - | 1.08E-11 |
| GO_BP | Enzyme linked receptor protein signaling pathway | - | - | 2.06E-10 |
| GO_BP | Positive regulation of gene expression | - | - | 5.75E-08 |
| GO_BP | Positive regulation of transcription, DNA-dependent | - | - | 5.04E-08 |
| GO_BP | Tissue morphogenesis | - | - | 2.78E-08 |
| GO_BP | Embryonic morphogenesis | - | - | 2.43E-08 |
